# Experimental and Numerical Study of Radioiodine Sorption and Transport in Hanford Sediments

**DOI:** 10.1021/acsearthspacechem.3c00291

**Published:** 2024-01-16

**Authors:** Xiaoliang He, Mark L. Rockhold, Yilin Fang, Amanda R. Lawter, Vicky L. Freedman, Rob D. Mackley, Nikolla P. Qafoku

**Affiliations:** † Pacific Northwest National Laboratory, Richland, Washington 99354, United States; ‡ Sealaska Technical Services, Richland, Washington 99354, United States; § Department of Civil and Environmental Engineering, University of Washington, Seattle, Washington 98195, United States

**Keywords:** radioiodine, transport, sorption, ferrihydrite, distribute-rate model

## Abstract

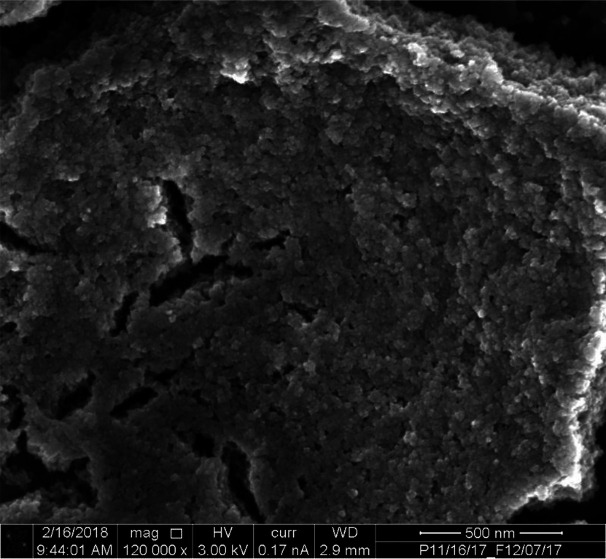

Radioiodine (^129^I) poses a potential risk to human health and the environment at several U.S. Department of Energy sites, including the Hanford Site, located in southeastern Washington State. Experimental studies and numerical modeling were performed to provide a technical basis for field-scale modeling of iodine sorption and transport behavior. The experiments were carried out using six columns of repacked contaminated sediments from the Hanford Site. Although iodate has been determined to be the dominant iodine species at the Hanford Site, the sorption and transport behaviors of different iodine species were investigated in a series of column experiments by first leaching sediments with artificial groundwater (AGW) followed by AGW containing iodate (IO_3_^–^), iodide (I^–^), or organo-iodine (2-iodo-5-methoxyphenol, C_7_H_7_IO_2_). Ferrihydrite amendments were added to the sediments for three of the columns to evaluate the impact of ferrihydrite on ^129^I attenuation. The results showed that ferrihydrite enhanced the iodate sorption capacity of the sediment and retarded the transport but had little effect on iodide or organo-I, providing a technical basis for developing a ferrihydrite-based remedial strategy for iodate under oxidizing conditions. Data from the column transport experiments were modeled using the linear equilibrium Freundlich isotherm model, the kinetic Langmuir adsorption model, and a distributed rate model. Comparisons of the experimental data and modeling results indicated that sorption was best represented with the distributed rate model with rates and maximum sorption extents varying by iodine species and ferrihydrite treatment. However, the linear Freundlich isotherm (*K*_*d*_) model was also found to fit the laboratory experimental data relatively well, suggesting that the *K*_*d*_ model could also be used to represent iodine transport at the field scale.

## 1 Introduction

Radioiodine (^129^I) has long been recognized as an environmental concern because it is toxic, has a very long half-life (15.7 million years), and is highly mobile in groundwater.^1^ Radioiodine can pose a risk to public health because it passes through the food chain and can accumulate in the human body, mostly in the thyroid gland, thereby increasing the risk of thyroid cancer.^2^ Radioiodine plumes exist at two major U.S. Department of Energy (DOE) sites, including the Hanford Site, located in southeastern Washington, and the Savannah River Site in South Carolina. Radioiodine was generated at Hanford as a nuclear fission product of uranium and plutonium and entered the subsurface through intentional and unplanned releases of liquid wastes into the soil, followed by percolation of the wastes into the underlying groundwater.^3^ Evaluating the transport and fate of radioiodine at Hanford and elsewhere is important for risk assessment, site management, and remediation.

**Figure 1:**
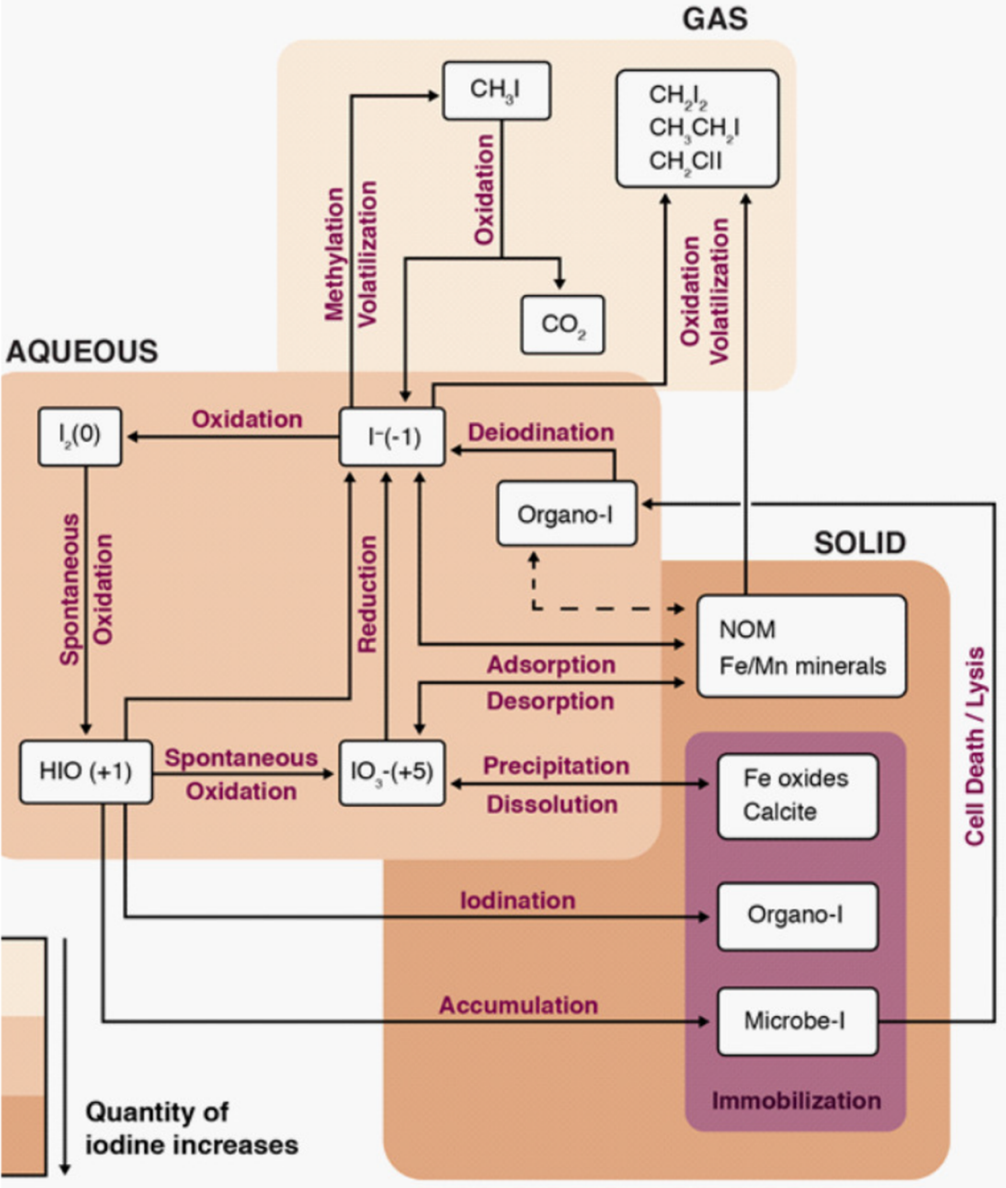
Biogeochemical process framework for iodine in the subsurface environment involving species interactions in three phases: solid, aqueous, and gas. Reprinted with permission from Neeway et al.^1^ Copyright 2019 Elsevier.

Uncertainties in the transport and fate of radioiodine at Hanford exist due to uncertainties about the volumes and timing of historical waste discharges, variable compositions of disposed waste, heterogeneity of the physical and hydraulic properties of the subsurface, and uncertain biogeochemical reaction processes.^3^ The ability to reliably predict the transport and fate of radioiodine through the subsurface is needed for risk assessment and remedy evaluation. Neeway et al.^1^ reviewed characteristics of the Savannah River and Hanford Sites and developed a biogeochemical process framework to describe how iodine species and transformation processes are controlled under the prevailing biogeochemical conditions (see Figure 1). Hu et al.^4^ performed integrated column and batch experiments to study the sorption and transport behaviors of iodate, iodide, and 4-iodoaniline in sediments from both Savannah River and Hanford, and found distinct transport characteristics between species.

The behavior of iodine in the environment is complex due to multiple physical and redox states, interactions with organic matter, and microbial transformations.^3^ Iodine can form hypodiodous acid in water, and both species can react with natural organic matter to form organo-I compounds.^5^ Hanford sediments generally have a very low natural organic matter content^6,7^ and relatively low microorganism densities, except in the hyporheic zone along the Columbia River.^6,8,9^ However, various organic compounds are known to have been codisposed with radioiodine and other contaminants at Hanford waste disposal sites, as discussed by Truex et al.^3^ Microbial abundance is high enough to effect biomineralization as reported in Neeway et al.,^1^ Fredrickson et al.^10^ and Szecsody et al.^11^ Stable iodine-127, which exhibits the same chemical behavior in the subsurface as iodine-129, is found at much higher concentrations in Hanford groundwater, with a ratio of ^127^IO_3_^–^ to ^129^IO_3_^–^ estimated at 1000 to 1.^3^ Although the source of iodine-127 is uncertain, iodine is known to be a trace constituent of nitric acid, which was used in large quantities during Hanford operations. Iodine-127 participates in the same biogeochemical processes as iodine-129, and current remediation technologies are not specific for a particular iodine isotope.^2,3^ Therefore, the proportion of iodine-127 to iodine-129 needs to be considered to avoid costly treatment of stable iodine^1^ for any in situ radioiodine remedy. There are currently no technologies available for treating radioiodine in groundwater. Therefore, the interim remedy for the radioiodine plume in the 200-UP-1 operable unit is hydraulic control via injection wells that are part of a pump-and-treat (P&T) well network in the Hanford 200 West Area.^12^

The liquid wastes were disposed on the ground surface and infiltrated through the Hanford vadose zone to the groundwater and were subjected to sorption, precipitation, and volatilization as shown in Figure 1. Groundwater monitoring results indicate that ^129^I groundwater plumes at Hanford have been slowly but steadily shrinking since the early 1990s,^3,13^ but large areas still exist with concentrations that exceed 1 pCi/L, the maximum concentration level allowed by federal and state regulations. Volatilization is likely a relatively minor mechanism responsible for the observed groundwater plume attenuation, but this process would be more significant in the vadose zone owing to the presence of an interconnected gas phase.^14^ The exact mechanisms for the attenuation of radioiodine groundwater plumes over time at Hanford are unknown, but sorption has been assumed to be the dominant process.

Iodine association with natural organic matter is important in sediments, even when organic carbon concentrations are very low (e.g., ≤0.2% at the Hanford Site).^15−17^ Xu et al.^17^ performed sequential extractions on Hanford sediment samples and showed that a substantial fraction of sediment-associated iodine was more strongly bound to sediments than expected. Organic carbon appeared to control iodine binding to sediments and was assumed to be responsible for the incorporation of residual iodine (57.1% to 90.6%). Xu et al.^17^ also showed that the higher the organic carbon concentrations in the sediments, the higher the values of *K*_*d*_ for both adsorption and desorption, and the higher the residual iodine concentrations.

Metal oxides and hydroxides (e.g., Fe(OH)_3_, Al(OH)_3_, MnO_2_) may also play an important role in controlling iodine behavior in soils, through both adsorption of inorganic iodine and oxidation of iodide.^18,19^ Ferric, aluminum, and manganese oxides have been shown to adsorb iodate more strongly than iodide.^20−22^ Wang et al.^23^ performed a series of macroscopic experiments to estimate the effectiveness of iodate and iodide adsorption on four different Fe oxides (ferrihydrite, goethite, magnetite, and hematite) at various pH levels and solution ionic strengths. Among them, ferrihydrite showed the greatest potential to become a practical sorbent for iodate. Similar conclusions have also been reported by Pearce et al.^24^

The dominant aqueous iodine species in the vadose zone at Hanford were reported to be iodate (84–98%), iodide (1–2%), and organo-iodine (0–14%) .^16,25−27^ Early studies of iodide sorption reported values of *K*_*d*_ in the range of 0 to 2 mL/g,^28^ but more frequently between 0 and 0.2 mL/g.^29^ More recently, studies in Hanford sediments have identified species-specific sorption behavior, with *K*_*d*_ values ranging from 0.3 to 1.2 mL/g (retardation factor of 2.4 to 6.2) for iodate, and from 0.07 to 0.1 mL/g (retardation factor 1.3 to 1.5) for iodide.^3,11^ The desorption *K*_*d*_ values have been found to be higher than the values of iodine adsorption *K*_*d*_, indicating that the sorption is only partially reversible.^17^ Similar trends have been observed for iodide and iodate, indicating iodate sorption to Hanford Site sediments was greater than iodide sorption.^3,20^

In the present study, a series of laboratory experiments was performed in sediment-packed columns saturated with water to further characterize and model the transport and sorption/desorption behavior of three aqueous iodine species: iodate (IO_3_^–^), iodide (I^–^), and organo-iodine (2-iodo-5-methoxyphenol) to support field-scale modeling predictions of radioiodine transport and fate in groundwater. Experiments were performed with and without ferrihydrite amendments to evaluate its ability to naturally attenuate radioiodine in Hanford sediments. The linear equilibrium Freundlich isotherm model, kinetic Langmuir adsorption models, and a distributed rate (DR) model^30^ using the Langmuir model were evaluated for their ability to represent observed radioiodine sorption behavior for iodide, iodate, and organo-iodine. The experimental procedure and numerical models are detailed in the following section.

## 2 Methodology

### 2.1 Laboratory Experiments

**Figure 2:**
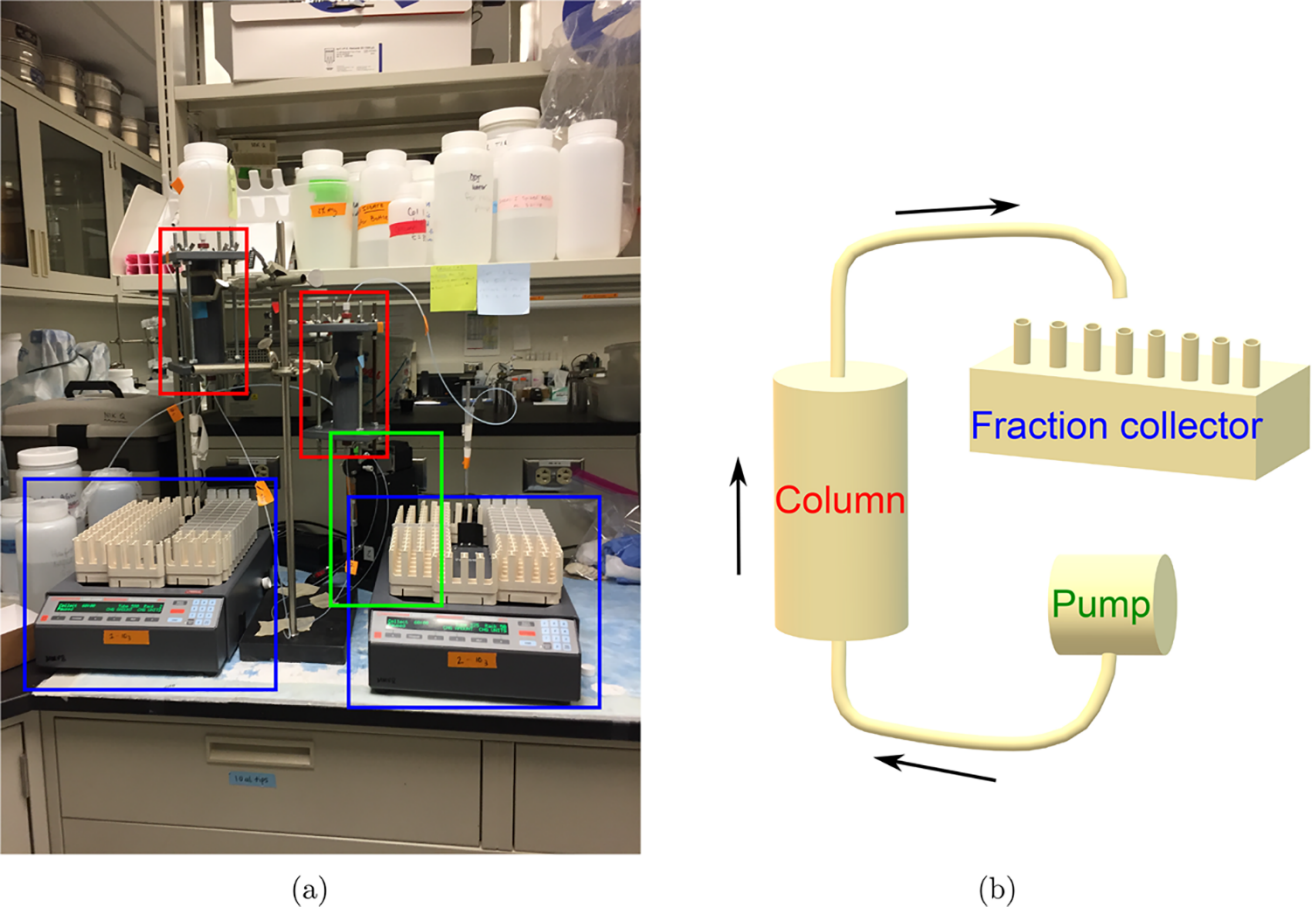
(a) Column experiment setup, where the red rectangles are columns 1 and 2, the blue ones are two fraction collectors, and green is the pump used to push water through the columns from bottom to top. (b) The schematic of the experimental setup.

Laboratory experiments were performed using 14.4 cm-long by 3.2 cm-diameter poly(vinyl chloride) (PVC) columns packed with the sieved ≤2 mm size fraction of contaminated field sediments collected from the 200-UP-1 groundwater operable unit, located on the Central Plateau at Hanford, the region with the highest radioiodine groundwater concentrations (∼50 pCi/L) .^20^ Although detailed mineralogy analysis was not performed for this batch of sediments, Serne et al.^31^ conducted a comprehensive data review on the mineralogy of Hanford Central Plateau sediments. The overall mineral composition for sand fractions is dominated by quartz (∼66% to 82% by mass) and feldspars (∼15% to 31%). Similar observations were obtained for the silt fraction (∼61% to 76% of quartz and ∼19% to 44% of feldspars). However, the clay fractions were dominated by illitic mica (∼42% to 60%) and chlorite (∼14% to 17%). The sediments were stored at 4 °C in the laboratory under air-dry conditions for approximately 2 weeks prior to use. The experiments were carried out under water saturated conditions, with and without ferrihydrite added to the sediments, to characterize the transport and sorption/desorption behavior of three species of aqueous iodine: iodate (IO_3_^–^), iodide (I^–^), and organo-iodine (2-iodo-5-methoxyphenol). 2-Iodo-5-methoxyphenol was selected as the organo-iodine in the current study because this organic compound was believed to have a structure similar to that of the species from field samples, i.e., the presence of the phenol group and the direct coordination of the iodine atom with carbon. Figure 2(a) shows the setup of the column experiments conducted in the laboratory, and (b) demonstrates the conceptual schematic diagram. Table 1 lists the experimental variables for each of the columns. The iodide concentration is 90 μg/L in the influent while both iodate and organo-I are 100 μg/L. This was unintentional. However, the impact to the overall results was not significant, as discussed in the modeling results section.

**Table 1:** Conditions for All Six Columns, Where φ Is the Porosity, *V*_*D*_ Is the Flow Velocity, and *C*_0_ Is the Species Concentration in AGW

column	φ	*V*_*D*_ (cm/h)	species	*C*_0_ (μg/L)	ferrihydrite amended
1	0.47	0.65	IO_3_^–^	100	No
2	0.43	0.66	IO_3_^–^	100	Yes
3	0.47	0.67	I^–^	90	No
4	0.44	0.66	I^–^	90	Yes
5	0.45	0.64	Organo-I	100	No
6	0.44	0.63	Organo-I	100	Yes

As shown in Figure 2(b), influent with and without iodine was pumped into the sediment-packed columns through the bottom. The effluent flowing from the tops of the columns was collected in a fraction collector in 7 mL vials (5 mL sample size) that were capped after the effluent collection. Transport experiments were performed using iodate in columns 1 and 2, iodide in columns 3 and 4, and organo-I in columns 5 and 6. To evaluate the influence of enhanced sorption on iron oxides, the sediments used in three of the columns (2, 4, and 6) were amended with ferrihydrite prior to packing. The ferrihydrite used in the experiment was generated following a well-established method described by Qafoku et al.^20^ Note that to better present the results and conduct comparisons, the ordering of the six columns is rearranged from what was reported^20^ previously. The mass fraction of ferrihydrite added to each of these three columns was 1%. Four steps were followed to add ferrihydrite into the sediments as follows: (1) 200 g of the <2 mm size fraction separated from the sediment; (2) ferrihydrite was added as a slurry (about 2.44 g); (3) it was thoroughly mixed with fine grains; (4) well-mixed sediment with ferrihydrite ready to use. The columns were leached with AGW with a chemical composition listed in Table 2. The reagents were added to the double deionized water, according to the listing order. Once the chemicals were dissolved, an excess of calcium carbonate (CaCO_3_) was added to the solution. AGW was stirred and kept open to the air for approximately 1 week until the pH reached a value of about 7.5. Subsequently, excess CaCO_3_ in the solution was filtered out using a 0.45-μm filter.

**Table 2:** Artificial Groundwater Recipe^32^

constituent	*C* (mg/L)
H_2_SiO_3_·*n*H_2_O, silicic acid	15.3
KCl, potassium chloride	8.20
MgCO_3_, magnesium carbonate	13.0
NaCl, sodium chloride	15.0
CaSO_4_, calcium sulfate	67.0
CaCO_3_, calcium carbonate	150.0

The column experiments were performed in three phases:

- Phase 1: Sediment-packed columns were first leached with AGW without iodine until the iodine concentrations in the effluent either did not change or were below the detection limit of the instrument (0.126–1.26 μg/L, depending on sample dilution). About 35 pore volumes of iodine-free solution was passed through each column in this phase.
- Phase 2: The iodine-spiked AGW was then injected at the bottom of the columns to identify the adsorption behaviors of different species of iodine. The species of iodine used in the AGW for each column are listed in Table 1.
- Phase 3: The columns were leached with iodine-free AGW, similar to phase 1, to characterize the desorption behavior of iodine.

The main objective of this study is to characterize the sorption–desorption behavior of different iodine species in Hanford Site sediments. Phase 1 was a preliminary procedure that was intended to remove the most mobile iodine species from the sediment. As a result, the emphasis is placed on phases 2 and 3. The observational and numerical data from those two phases are presented and discussed in the following sections.

The flow of the influent solutions was intentionally stopped multiple times (in stop-flow events) to evaluate the kinetic sorption and mass transfer effects. The number of stop-flow events for columns 1 and 2 was 10; the rest of the columns had 5. Effluent samples were collected periodically throughout the experiments to measure the concentration of iodine and the concentrations of major ions. Total aqueous iodine was routinely measured in all phases of the experiments to determine the amount of iodine sorbed onto the sediments as reported.^20^ Data for phases 2 and 3 are reported here to quantify the sorption and desorption behavior. Phase 1 of the column experiments is considered a preliminary step and is not described in this manuscript. The details are provided by Qafoku et al.^20^ It is noteworthy to mention that the field conditions are expected to be generally much more variable than the experimental conditions used in the laboratory. The sediments used in the laboratory and the experimental conditions were chosen to provide baseline measurements under more controlled conditions to better isolate the effects of ferrihydrite on iodine sorption without many of the confounding factors that can influence the transport of contaminants in the field. Lastly, no unexpected or unusually high safety hazards were encountered in the experiments.

### 2.2 Numerical Simulation

#### 2.2.1 Numerical Solver and Model Setup

Numerical simulations of water flow and solute transport were performed using the STOMP simulator.^33^ The governing equations in STOMP are discretized using an integral-volume finite difference method^34^ with backward Euler time differencing. Nonlinearities in the discretized equations are resolved through a Newton–Raphson iteration. Multicomponent reactions associated with biogeochemical processes are solved using coupled equilibrium, conservation, and kinetic equations.^35,36^ For mobile species, the reaction equations are solved after solving for advection and dispersion using an operator-splitting approach. In this study, the flow of water and the transport of iodine in the columns were simulated using a 1D model with the length of the column divided into 50 grid blocks of equal size. The water flow rates and iodine concentrations were prescribed for the lower boundary, the inlet end of the numerical model, to match the experimental conditions. Aqueous pressures for flow and outflow conditions for transport were prescribed for the upper boundary, the outlet end of the model. Flux-average iodine concentrations exiting the top of the model domain were computed from iodine mass flux and water volumetric flow rates and compared with observational data.

#### 2.2.2 Transport Models

Contaminant transport in the subsurface is generally represented by using the advection-dispersion-reaction equations. The simplest and most widely applied approach for modeling sorption is the one-parameter linear equilibrium Freundlich isotherm model. The distribution coefficient, *K*_*d*_, is assumed to be a constant value for any specific porous medium and solute. When nonlinear relationships between sorbed and aqueous concentrations, and/or finite sorption capacities, are observed, nonlinear Freundlich or Langmuir sorption models are often used.^37^ Kinetic forms of these models allow for observed rate- and/or process-dependent (e.g., adsorption–desorption) effects if needed. A distributed rate model based on the kinetic Langmuir model was also implemented. The DR model is constructed based on the assumption that multiple sorption sites exist simultaneously and contribute to the overall reaction according to their individual kinetic properties. Qafoku et al.^38^ used a similar method to model uranyl adsorption and desorption in a Hanford Site sediment. A brief review of these models is provided in the following.

The linear equilibrium Freundlich isotherm model assumes a linear relationship between the absorbed solute concentration (*s* [mol/kg]) and the aqueous solute concentration (*c* [mol/m^3^]) by the constant distribution coefficient (*K*_*d*_). The mathematical expression is written as
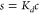
1

The kinetic Langmuir model^39^ (eq 2) states that the sorption rate (dθ/d*t*) is the difference between the adsorption and desorption rates. The adsorption rate is determined by the concentration of the aqueous species (*c*), the available adsorbent surface (1 – θ), and the adsorption rate coefficient (*k*_ad_). The desorption rate is the product of the adsorbed amount (θ) and the desorption rate coefficient (*k*_de_). θ is nondimensionalized as eq 3, where *a* [mol/kg] is the actual adsorbed amount and β [mol/kg] is the total sorption capacity. Therefore, three parameters (*k*_ad_, *k*_de_, and β) must be specified to use the Langmuir kinetic Langmuir model.
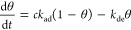
2
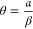
3

For the DR model, multiple sorption sites were specified (*m* = 45) for all columns in this study. The mass transfer between the aqueous and solid phases at each site was described by a kinetic Langmuir model; that is, each site exhibits distinct kinetic properties. Thus, a series of Langmuir parameters (*k*_ad_^*i*^, *k*_de_^*i*^, and β^*i*^) are needed to represent the sorption/desorption behaviors at each site (eq 4). Similar to the model setting described by Liu et al.,^30^*k*_ad_^*i*^ was assumed to be proportional to a constant *K* and *k*_de_^*i*^ (eq 5). The total sorption capacity (β) is uniformly distributed over all sites (eq 6). To further reduce the dimensionality of the model, forward rates (*k*_ad_^*i*^) were set to follow a log-normal distribution^30,38^ as formulated by eq 7, where *p* is the probability of a site with sorption coefficient *k*_ad_^*i*^. The log-normal distribution is defined by the logarithm mean rate μ and standard deviation σ. In addition to those two parameters, the DR model requires two more inputs, β and *K*, for a total of four model parameters.
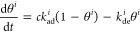
4
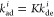
5
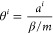
6
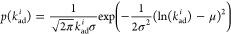
7
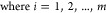


#### 2.2.3 Reaction Networks

Concentrations of specific iodine species were measured mainly during phase 1 of the experiments and only sporadically during phases 2 and 3, due to difficulty in the measurement procedure. However, total iodine concentrations in the column effluent were measured routinely during phases 2 and 3. Field data indicate that iodate is the dominant species in Hanford groundwater and that iodate sorbs more strongly than iodide. Therefore, the following simplified reaction network was developed for modeling the column experiments:
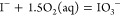
8
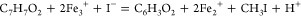
9
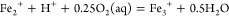
10

Eq 8 is an O_2_-dependent equilibrium speciation reaction for iodate and iodide, where the equilibrium constant (log(*K*)) is 18.116.^40^Eqs 9 and 10 are pH-dependent redox reactions that explain the transformation of organo-I to iodomethane (CH_3_I) following one of the two mechanisms proposed by Keppler et al.^41^ In eq 9, which was modeled using a forward–backward rate equation with rates estimated by inverse modeling, organo-I is not shown explicitly but is dissociated into species C_7_H_7_O_2_ and I^–^. Although the experimental and modeled systems were fully water saturated, in a two-phase (air–water) system, the volatile species iodomethane could partition into and diffuse in the gas phase, providing a potential mechanism for loss of iodine near the ground surface to the atmosphere. The equilibrium constant (log(*K*)) for the reaction delineated in eq 10 is 8.779.^40^

#### 2.2.4 Parameter Estimation

Model parameters were estimated with the parameter estimation code PEST,^42^ using an objective function that minimized the differences between the observed and simulated total iodine concentrations from the column effluent. Model performance was evaluated by visual inspection of observed and simulated breakthrough curve (BTC) results, and by comparison of the goodness-of-fit metric root-mean-square error (RMSE) following the formulation with some modification by Barnett et al.:^43^
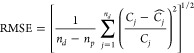
11where *n*_*d*_ is the number of data points, *n*_*p*_ is the number of input parameters, *C*_*j*_ and  are the observed and simulated concentrations. *C*_*j*_ was used to normalize the difference instead of the initial concentration^43^ (*C*_0_) because there exists a significant magnitude variance (up to 10^2^) in the data sets. RMSE can be considered as a measure of the variance between the simulated and observed data. The model with the lowest RMSE value is considered the best. To account for the experimental uncertainty in the parameter estimation procedure, two different weighting schemes were tested in the optimization procedure. The first scheme assigned a uniform weighting factor of 1 to all observational data, and the second one used an inverse approach to determine the weighting factor; i.e., the weighting factor (WF) for each observational data is calculated as the ratio between the iodine concentration in the influent and the measured values in the effluent,
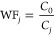
12

The equilibrium *K*_*d*_ model provided similar predictions regardless of the two weighting schemes. But the kinetic and DR models generated significantly better fittings to the observational data when using the weighting scheme described in eq 12. Thus, the *K*_*d*_ model used the uniform weighting factor, while the inverse method was applied to other models in the present study.

## 3 Results and Discussion

**Figure 3:**
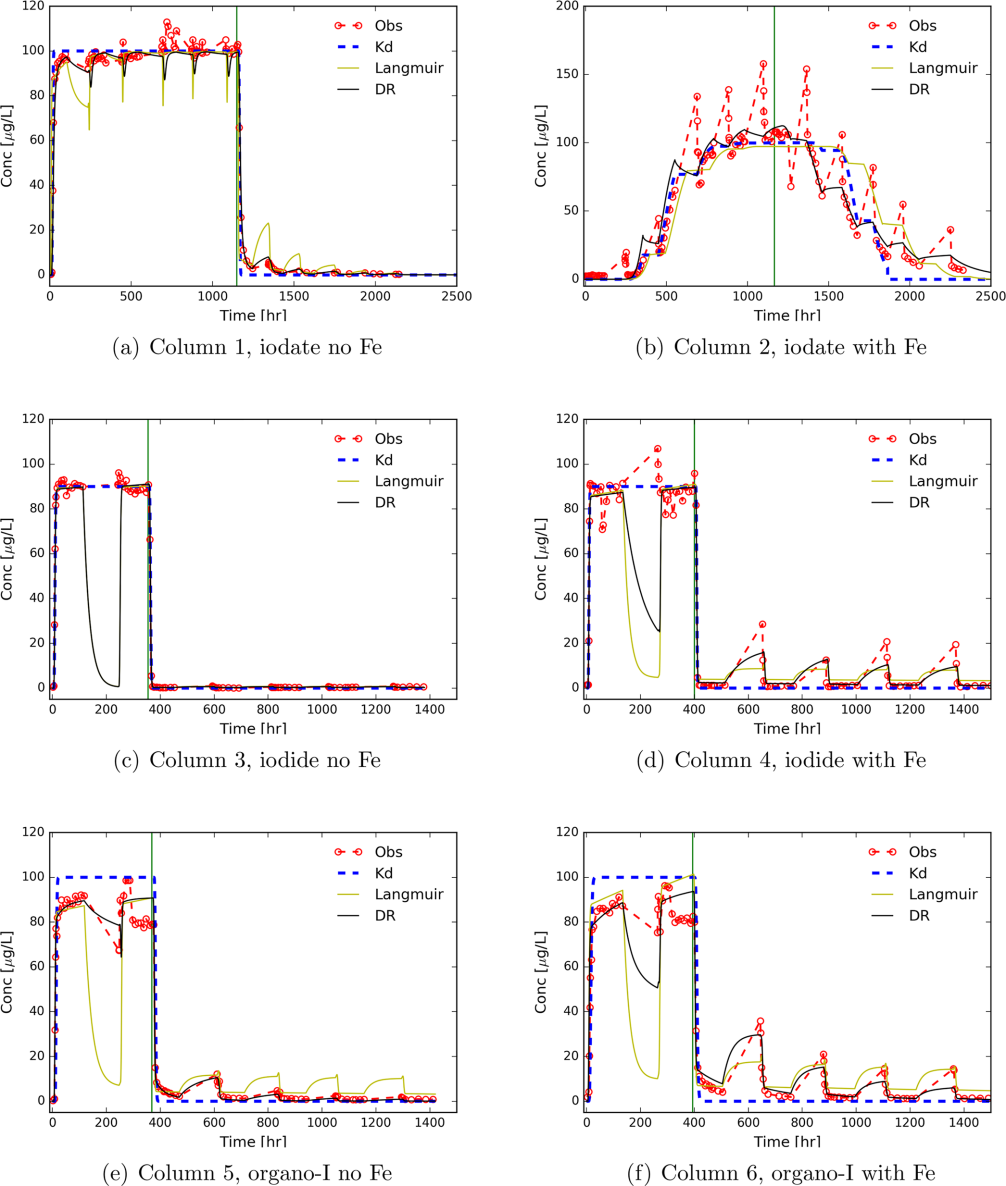
Comparisons of iodine concentration in effluent measured at the top of each column with both observational data and modeling results. The results from columns without ferrihydrite are on the left-hand side; results with ferrihydrite are on the right-hand side. Panels (a) and (b) are from columns with influent spiked with iodate, (c) and (d) with iodide, (e) and (f) with organo-I. The green vertical line denotes the initiation of phase three (leaching with iodine-free AGW).

**Figure 4:**
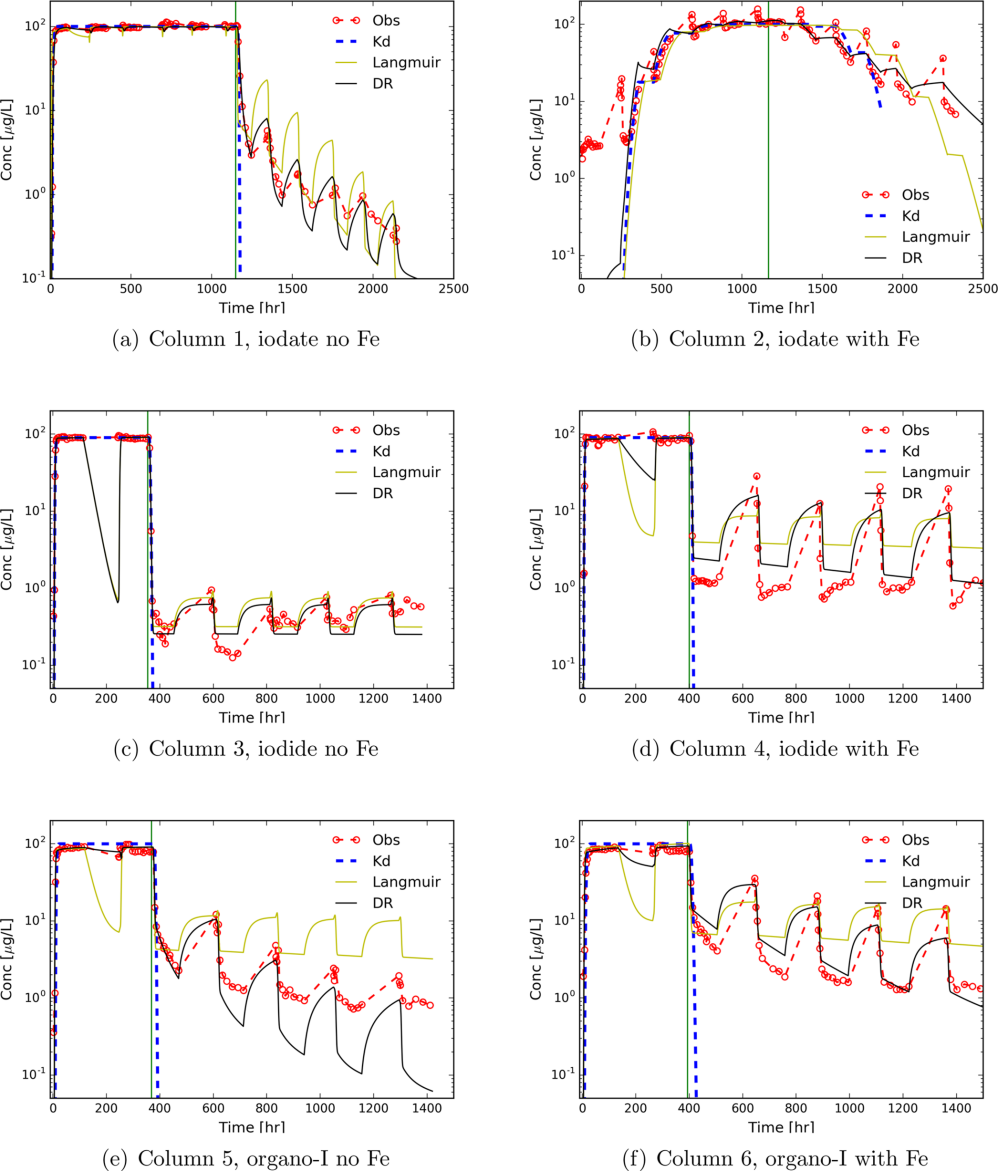
Comparisons of iodine concentration in effluent measured at the top of each column with both observational data and modeling results. This figure shows the same data as what are plotted in Figure 3, except the *y* axis is in log scale. The green vertical line denotes the initiation of phase three (leaching with iodine-free AGW).

Observed and simulated breakthrough curves for phases 2 and 3 of the experiments are shown in Figures 3 and 4, where the same data were plotted using linear and logarithmic scales for the *y* axis, respectively. In both figures, the abscissa is time [h] and the ordinate is the total iodine concentration (Conc [μg/L]). To better illustrate the influence of ferrihydrite amendments and different iodine species on sorption/desorption processes, Figures 3 and 4 are organized with the results with native sediments (columns 1, 3, and 5) on the left and ferrihydrite amended sediments (columns 2, 4, and 6) on the right. The plots in the first, second, and third rows represent the experiments performed with iodate, iodide, and organo-I, respectively. A vertical green line in each figure denotes the ending of leaching with iodine-spiked AGW and the starting with iodine-free AGW. The fitted parameters for each model are reported in Table 3. Table 4 lists the goodness-of-fit metric RMSE used to evaluate the fit of the models.

**Table 3:** Parameters Estimated by Inverse Modeling for the Sorption Reactions in Six Column Experiments^a^

		column					
		1	2	3	4	5	6
model	parameter	IO_3_^–^	IO_3_^–^ (Fe)	I^–^	I^–^ (Fe)	organo-I	organo-I (Fe)
*K*_*d*_	*K*_*d*_ [mL/g]	0.256	8.640	1.010 × 10^–4^	1.935 × 10^–3^	1.388 × 10^–4^	2.120 × 10^–4^
							
Langmuir	*k*_ad_ [1/s]	8.561	116.663	8.997 × 10^–2^	0.167	0.442	1.084
	*k*_de_ [1/s]	5.357 × 10^–3^	0.802	2.874 × 10^–5^	3.430 × 10^–4^	5.021 × 10^–4^	6.700 × 10^–4^
	β [mol/kg]	4.880 × 10^–7^	7.620 × 10^–5^	1.375 × 10^–4^	8.411 × 10^–5^	2.422 × 10^–2^	1.198 × 10^–2^
							
DR	μ	–24.275	–6.057	–25.800	–23.796	–2.155	–23.664
	σ	8.686	4.174	6.700	6.495	0.780	6.461
	β [mol/kg]	4.055 × 10^–5^	5.387 × 10^–5^	9.240 × 10^–4^	6.908 × 10^–5^	9.458 × 10^–6^	1.463 × 10^–4^
	*K* [mL/g]	8.654	1.392	7.440 × 10^–2^	0.653	22.098	6.338

a (Fe) means this column is amended by ferrihydrite.

**Table 4:** Goodness-of-Fit Metric and Model Evaluation Criteria^a^

column	influent	model	RMSE
1	IO_3_^–^	*K*_*d*_	0.529
		Langmuir	6.398
		DR	0.290
			
2	IO_3_^–^(Fe)	*K*_*d*_	0.573
		Langmuir	0.638
		DR	0.569
			
3	I^–^	*K*_*d*_	0.794
		Langmuir	0.538
		DR	0.452
			
4	I^–^ (Fe)	*K*_*d*_	0.780
		Langmuir	2.229
		DR	1.535
			
5	Organo-I	*K*_*d*_	0.919
		Langmuir	2.190
		DR	0.712
			
6	Organo-I (Fe)	*K*_*d*_	0.825
		Langmuir	1.478
		DR	0.659

a (Fe) means the column is amended by ferrihydrite.

### 3.1 Observational Data

Figures 3 and 4 show that the measured iodine adsorption was relatively small for most experiments and that the breakthroughs were achieved quickly (about 20 to 30 h) after the injections of AGW. Column 2 is an exception in that iodate-spiked AGW was injected into the ferrihydrite-amended soil and exhibited significant retardation of iodate transport in both sorption and desorption phases. Figures 3(a,b) and 4(a,b) illustrate that the iodate transport was significantly retarded due to the addition of ferrihydrite in the sediment but not iodide or organo-I. The overall behaviors of transport with iodide and organo-I did not exhibit major changes in the presence and absence of ferrihydrite. This is consistent with findings in previous studies that showed ferrihydrite to be the most efficient Fe oxide for removal of iodate from aqueous solution,^23,24^ with slow desorption relative to adsorption.

The ferrihydrite amended sediment has a slightly acidic pH. The pH for the effluent from columns with ferrihydrite was measured to be around 6.5 in phase 1. Because of the slightly acidic pH created in the mixture sediment-ferrihydrite, dissolution of sediment carbonates was promoted in this system. This was confirmed by the measurements of the Ca, Mg, and Ba effluent concentrations performed in the first pore volume, which were much greater in the columns amended with ferrihydrite compared to the columns that did not have ferrihydrite. As a result, some iodine may have been released during the carbonate mineral dissolution in the ferrihydrite columns. Iodine released from carbonate minerals is expected to be iodate. However, data from the iodine speciation analyses conducted in the effluent samples confirmed that the speciation of iodine in the experiment with no ferrihydrite was split between iodate and iodide, while iodine speciation was dominated by iodide in the effluents of columns with ferrihydrite in the first pore volume.

Iodine concentrations were spiked during and after stop-flow events. This was more heavily pronounced in the columns amended with ferrihydrite (right-hand side of Figures 3 and 4) for all three iodine species. This behavior potentially results from adsorption/desorption time dependence on the multisite sorption, multidomain mass transfer processes, and/or other reactions occurring simultaneously. The exact mechanism is unclear^24^ and requires further investigation. Moreover, the magnitude of the total iodine concentration in column 2 during the third to sixth stop-flow events is about 1.5 times higher than in the injected AGW. This is a strong indication that some amount of iodine still resided in the sediment after leaching in phase 1 of the experiments. The added iron oxides may have triggered the release of iodine from the sediments. Again, a fundamental understanding of such processes is lacking and requires future investigation.

It is noteworthy that the total iodine concentration did not reach a full breakthrough (that is, *C* = *C*_0_) when the source of iodine was organo-I, suggesting more complex adsorption or transformation reactions involving organo-I species. *C*_0_ is the total iodine concentration in the influent, which is 90 μg/L for iodide and 100 μg/L for iodide, and 100 otherwise. The simulated iodomethane concentration was found to be negligible (10 orders lower than the total iodine concentration), indicating that the influence of radioiodine volatilization should be minimal in water-saturated Hanford sediments.

### 3.2 Modeling Results

The fitted breakthrough curves generated from *K*_*d*_, Langmuir, and DR models are plotted in Figures 3 and 4 in addition to the observational data. Due to the magnitude difference of the total iodine concentration at sorption and desorption stages, the same data shown in Figure 3 are plotted again on a logarithmic scale in Figure 4 to better illustrate the evolution of the concentration. Visual inspection of the breakthrough curves shows that all three models tested can predict the BTCs for most of the columns reasonably well. Among them, the DR model provides the best fits, followed by the kinetic Langmuir model and then *K*_*d*_. This is an expected result since models with more degrees of freedom tend to produce better fits. The superiority of the DR model was largely demonstrated by the closer correspondence of observed and simulated BTCs during stop-flow events and its ability to better simulate the extended tailing of BTCs at later times. However, since no data were measured during the stop-flow events, it is difficult to calibrate the predicted results during those periods (as observed in column 2 and the first stop-flow event in column 4).

Among all six columns tested, column 2 exhibited distinct behavior, where not only the overall transport was significantly retarded, but also the behavior of sorption/desorption at stop-flow events was greatly changed. Consequently, even the more complicated DR model was not able to reproduce the observed BTC very accurately. The DR model responded to the stop-flow events but failed to reproduce the correct magnitude of the concentration spikes. As discussed previously, this may be due to the incomplete leaching of sorbed iodine from the contaminated sediment and an unknown iodine-releasing mechanism caused by ferrihydrite that was not present in the current model. For column 3, on the contrary, both the Langmuir and DR models performed similarly, suggesting that a relatively simple sorption–desorption relationship for iodide transport in the native sediment may be sufficient.

Figures 3 and 4 indicate that even the simplest *K*_*d*_ model can predict breakthrough curves that match the overall observations reasonably well. However, due to the equilibrium assumption of the *K*_*d*_ model, it cannot reproduce the effects of the stop-flow events and tailing. As shown in Table 3, the fitted *K*_*d*_ values for columns 3 to 6 are relatively small (<2 × 10^–3^ mL/g) but are 0.256 and 8.640 for columns 1 and 2, where iodate was injected into the native and ferrihydrite-amended sediments, respectively. Such behavior is consistent with the observation that ferrihydrite can greatly retard the transport of iodate, but has little effect on iodide and organo-I. In addition, the value of *K*_*d*_ (0.256) fitted for column 1 is in good agreement with what has been reported in the range of 0 to 1.2 mg/L^3,11,29^ for groundwater condition at Hanford, where iodate was reported to be the major iodine species.^44^ Therefore, although the DR model is the most capable model tested in the present study, the linear Freundlich model *K*_*d*_ could be adequate to simulate the transport of iodate in the column without ferrihydrite amendment and thus could be applicable to field-scale scenarios where the main iodine species is iodate and natural Fe oxides are less concentrated. Rockhold et al.^45^ used a *K*_*d*_ model to represent the transport of the plume ^129^I at the field scale and showed good agreement between the simulated and field-measured iodine concentrations. However, the results of the current study do show that the *K*_*d*_ model does not accurately reproduce the observed tailing of the BTCs from the column experiments (see Figure 4). Presumably, the two-parameter nonlinear model^46,47^ could be used to improve the prediction in future studies.

As previously pointed out in Table 3, an unintentional setting in the experiment caused the iodide concentration in the influent to be 90 μg/L, but both iodate and organo-I were 100 μg/L. However, the differences among iodine concentrations in the three input leaching solutions that had either iodide, iodate, or organo-I species in them are unlikely to significantly affect the overall results of this study. It is difficult to detect via experiments a significant effect of an increase (or decrease) in concentrations with 10 ppb on the extent and rate of iodine interaction with the sediments through different mechanisms. An additional simulation was performed for column 3 (leaching with iodide-spiked AGW in native sediments without ferrihydrite) using *K*_*d*_ model, where the influent concentration was adjusted to 100 μg/L with observational data scaled accordingly. The predicted *K*_*d*_ is in great agreement with that from a previous simulation with less than 0.1% difference.

**Figure 5:**
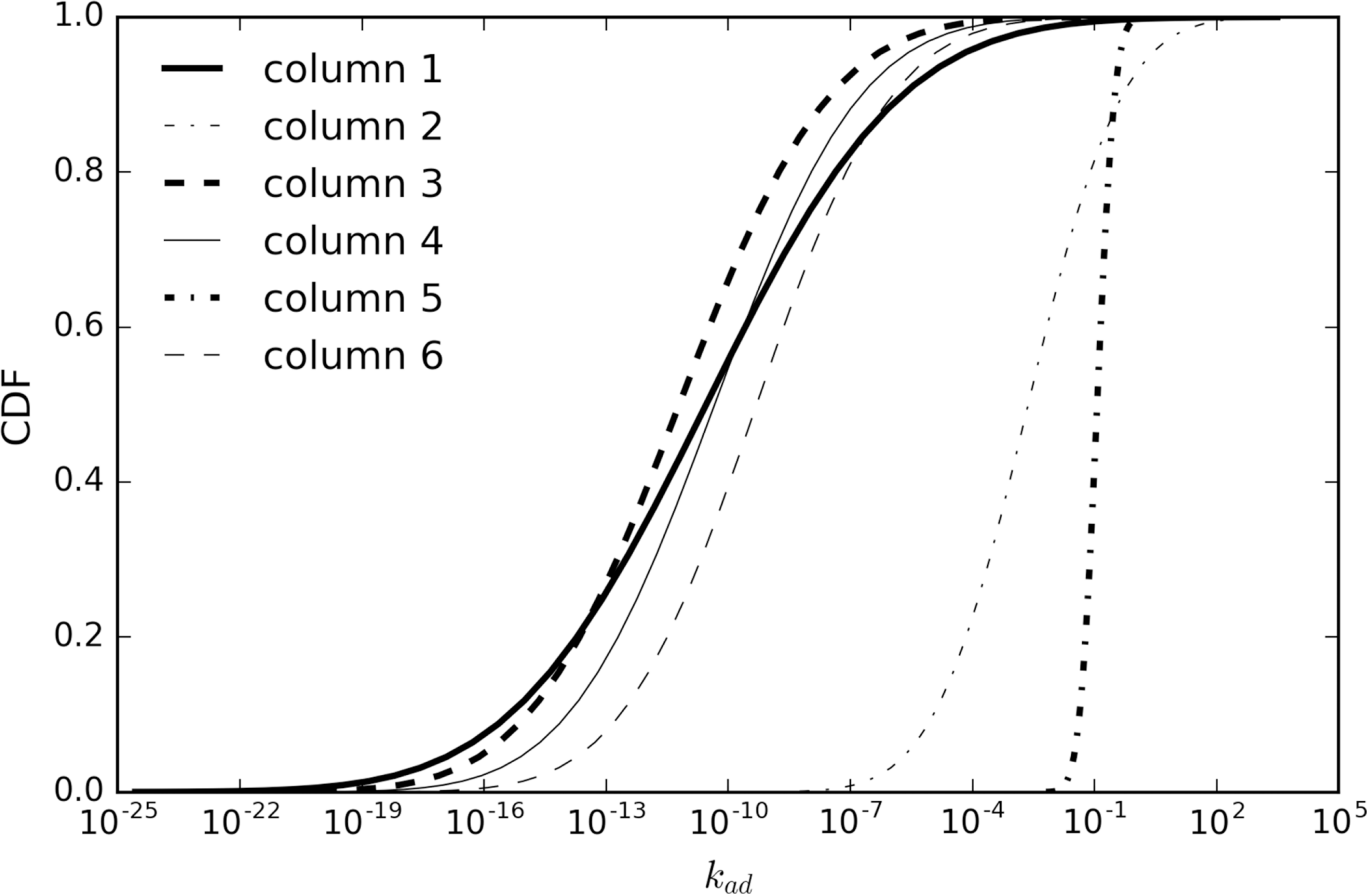
Cumulative probability function of the forward rates for the distributed rate model.

Similar to the *K*_*d*_ model, the adsorption and desorption rate coefficients (*k*_ad_ and *k*_de_) in the Langmuir models (116.663 and 0.802) are significantly higher for column 2 than those for the other column experiments. Furthermore, the maximum extent of adsorption (β) for the Langmuir kinetic model (7.62 × 10^–5^) is 2 orders of magnitude larger for column 2 (iodate with ferrihydrite) than that (4.88 × 10^–7^) for column 1 (iodate without ferrihydrite), suggesting the enhanced capacity of the sediment to adsorb iodate due to Fe oxide. To evaluate the fitted log-normal distribution functions, Figure 5 shows the cumulative probability functions (CDFs from 0.01% to 99.99%) associated with each column. The μ and σ reported in Table 3 were used to compute the CDFs. The forward rates for columns 1, 3, 4, and 6 ranged widely, from 10^–22^ to 10^–1^ but were limited to 10^–7^ to 10^2^ for column 2 and 10^–2^ to 10^–1^ for column 5. It is worth mentioning that the forward and backward rates for the reaction involving organo-I were found to be insensitive to the final results, which are 31.623 [1/s] and 0.101 [1/s] for column 5 and 1.476 [1/s] and 0.001 [1/s] for column 6.

The goodness of fit RMSE (Table 4) suggests that the DR model performs the best in the present study. As mentioned previously, after considering the number of degrees of freedom, the model with the lowest RMSE value is generally considered the best because it provides the closest fit to the observational data. It is interesting to note, however, that the RMSE values for the *K*_*d*_ model are actually lower than those for the Langmuir model for all but column 3. Furthermore, the RMSE value for the *K*_*d*_ model is lower than that for the DR model in column 4. This appears to be the result of spikes in iodine concentration that occurred during the early stop-flow events. The most significant improvement in prediction from using the DR model occurs in column 1, which can be observed in both Figures 3(a) and 4(a), and the RMSE values in Table 4. In addition, a substantial reduction in RMSE can be found for columns 4, 5, and 6. However, the RMSE values for Langmuir and DR models do not differ much for column 2 and column 3, but for different reasons. For column 2, the current model setup simply cannot reproduce the BTCs because of the lack of mechanistic understanding of the iodate sorption/desorption characteristics in Fe oxide-amended sediments, while the iodide transport is column 3 can be easily represented using the one-site assumption with the Langmuir model. This is consistent with the previous observations made in Figures 3 and 4. Since the same model settings were applied to all six columns, the RMSE values reported in Table 4 essentially suggest that the present models in this study are sufficient to predict iodate transport in native Hanford sediments as well as iodide and organo-I in both native and ferrihydrite-amended sediments. However, due to the limited fundamental understanding of iodate reaction mechanism and transport in ferrihydrite-amended sediments, further investigation may be needed.

## 4 Summary and Conclusion

Experimental and numerical investigations were performed to evaluate the iodine sorption and transport behavior in sediments from the U.S. DOE Hanford Site. Iodate, iodide, and organo-I spiked AGW was used to leach the native and ferrihydrite-amended Hanford sediments to model the sorption process, followed by iodine-free AGW to represent the desorption process. Ferrihydrite was found to significantly enhance sorption of iodate, consistent with previous studies,^23,24^ but had relatively little effect on iodide or organo-I. This provides a technical basis for developing a ferrihydrite-based remedial strategy for iodate under oxidizing conditions. Since ferrihydrite is a preferable sorbent for iodate, one possible remediation solution at Hanford is to inject the ferrihydrite in a well-mixed form into the capillary fringe and/or aquifer under known sources of contamination, which can adsorb the aqueous phase iodate and reduce the corresponding concentration in the groundwater. This would potentially provide an additional remedy that complements the P&T system that is designed for the treatment of other contaminants of concern.

To reproduce the observational data, an equilibrium *K*_*d*_ model, a kinetic Langmuir model, and a DR model were used to fit the experimental observations. The DR model was shown to provide the best fit to the results of the experiments, but the linear model was also able to generate a good fit to the data. However, the linear model could not reproduce the extended tailing of the experimental data. Although the DR model provided relatively good fits to the experimental data, more studies would be needed to better understand the mechanisms involved in the iodate mass transfer processes in sediments amended with ferrihydrite.

## 5 Quality Assurance

This work was performed in accordance with the Pacific Northwest National Laboratory (PNNL) Nuclear Quality Assurance Program (NQAP). The NQAP complies with DOE Order 414.1D, Quality Assurance. The NQAP uses NQA 1 2012, Quality Assurance Requirements for Nuclear Facility Application, as its consensus standard and NQA 1 2012 Subpart 4.2.1 as the basis for its graded approach to quality.

This work emphasized the acquisition of new theoretical or experimental knowledge. The information associated with this report should not be used as design input or operating parameters without additional qualification.
